# Deletion of *Alg13* disrupts postnatal development and migration of GABAergic cortical interneurons

**DOI:** 10.3389/fneur.2026.1757800

**Published:** 2026-03-31

**Authors:** Haibo Liu, Xin Qian, Hui Ma, Anhong Liu, Shuxiang Li, Xinyu Wang, Jing Zhang, Peng Gao

**Affiliations:** 1 Department of Neurosurgery, General Hospital of Ningxia Medical University, Yinchuan, Ningxia Hui Autonomous Region, China; 2 Ningxia Key Laboratory of Cerebrocranial Diseases, Ningxia Medical University, Yinchuan, Ningxia Hui Autonomous Region, China; 3 Department of Nerve Electrophysiology, General Hospital of Ningxia Medical University, Yinchuan, Ningxia Hui Autonomous Region, China; 4 Department of Pediatrics, General Hospital of Ningxia Medical University, Yinchuan, Ningxia Hui Autonomous Region, China; 5 Institute of Medical Sciences, General Hospital of Ningxia Medical University, Yinchuan, Ningxia Hui Autonomous Region, China; 6 Reproductive Medicine Center, General Hospital of Ningxia Medical University, Yinchuan, Ningxia Hui Autonomous Region, China

**Keywords:** *Alg13*, epilepsy, cerebral cortex, interneuron development, interneuron migration

## Abstract

**Background:**

Abnormal cortical neuron development is closely associated with various neurological disorders. Deletion of the *Alg13* gene has been identified as strongly associated with epilepsy susceptibility and seizure severity in mice. Similar deletions have also been observed in patients with epilepsy, indicating that *Alg13* may play a critical role in cortical interneuron development.

**Methods:**

Immunofluorescence analysis was used to assess the effects of *Alg13* deletion on the distribution and migration of interneurons in the cerebral cortex of postnatal mice. Transcriptome sequencing was performed to identify genes involved in neuronal development, and the findings were validated using reverse transcription–quantitative polymerase chain reaction (RT-qPCR).

**Results:**

Deletion of *Alg13* significantly influenced the spatiotemporal distribution of cortical interneurons in postnatal mouse brains. The migratory capacity of interneuron subtypes was markedly reduced in *Alg13*-deficient mice, indicating a potential increase in epilepsy susceptibility and seizure severity. Transcriptome sequencing and RT-qPCR validation identified three genes, *Ndn*, *Dynlt1b*, and *C3*, that were associated with the development of inhibitory interneurons.

**Conclusion:**

*Alg13* regulates postnatal interneuron development, and its deletion may contribute to epilepsy-related pathophysiology. These results enhance understanding of the molecular mechanisms underlying epilepsy and provide a potential experimental foundation and novel therapeutic targets for the management of *Alg13*-associated refractory seizures, thereby advancing knowledge of interneuron developmental regulation.

## 1 Introduction

Epilepsy is a group of neurological disorders characterized by recurrent seizures and represents one of the most prevalent chronic neurological conditions (1). Idiopathic epilepsy accounts for approximately 47% of all epilepsy cases and is believed to involve monogenic or polygenic inheritance patterns, affecting more than 700,000 individuals worldwide (2). Numerous pathogenic alterations contribute to seizures in epilepsy-related disorders, among which abnormalities in cortical neuronal development or function, resulting in an imbalance between cortical excitatory and inhibitory activity, are considered central pathogenic factors in epileptogenesis (3, 4).

Cortical function relies predominantly on two key neuronal populations: excitatory projection neurons and inhibitory interneurons (2). Although interneurons constitute only about 20 to 30% of cortical neurons, they are extensively distributed throughout the cerebral cortex (3). Impaired interneuron development has been associated with several neurodevelopmental disorders, including epilepsy (5). GABAergic interneurons are critical in the initiation and propagation of seizures by modulating cortical circuitry, synaptic transmission, neural network synchronization, and the balance between excitation and inhibition (6). Prior findings have shown that selective gene expression deficits in parvalbumin-positive (PV+) neurons were observed in epileptic cortical tissues exhibiting type I/III focal cortical dysplasia (7). Moreover, aberrant expression of neuropeptides such as somatostatin (SST) and neuropeptide Y (NPY) in various epilepsy models further emphasizes the importance of these interneuron subtypes in epilepsy pathogenesis (8). Although interneurons are essential for maintaining cortical function in epilepsy, the mechanisms underlying cortical interneuron development remain inadequately understood.

*UDP-N-acetylglucosaminyltransferase*, the enzyme encoded by the *Alg13* gene, plays a pivotal role in N-glycosylation, a process critical for protein folding, stability, and function. This enzyme catalyzes the transfer of an N-acetylglucosamine (GlcNAc) residue to glycoproteins, a modification that is essential for the proper functioning of many cellular processes, including neuronal migration and synaptic function. Disruptions in N-glycosylation, due to mutations in *Alg13* or other glycosylation genes, have been linked to various neurodevelopmental disorders, including epilepsy. In particular, MOGHE (mild malformation of cortical development with oligodendroglial hyperplasia) is associated with SL*C3*5A2 mutations, which impair the availability of galactose in the Golgi apparatus, an essential step for the proper glycosylation of proteins involved in neuronal development. Histopathological findings in MOGHE include heterotopic pyramidal neurons in the white matter, and experimental models of Sl*C3*5a2-deficient mice exhibit aberrant cortical neuron migration. Similarly, studies have shown that polysialylation of NCAM, a glycosylation process, plays a crucial role in neuronal migration, and aberrant N-glycosylation may perturb this process, contributing to migration defects in interneurons (9). Experimental findings have shown that *Alg13* deletion is associated with increased epilepsy susceptibility and greater seizure severity in mice (10). *De novo* mutations in *Alg13* have been identified in female patients presenting with severe intellectual disability and epilepsy, as well as in male infants exhibiting microcephaly, epilepsy, and early mortality due to congenital disorders of glycosylation (11, 12). Mutant mice with *Alg13 KO* showed increased seizure susceptibility attributed to disrupted neural circuitry, disruptions in neuronal development and cortical circuit organization are widely recognized as fundamental contributors to epileptogenesis through altered network excitability and excitation–inhibition balance (13). Additionally, previous observations revealed that *Alg13 KO* led to abnormal cortical architecture, altered neuronal density, and structural abnormalities in cortical neurons. Investigating the function and mechanism of *Alg13* in epilepsy, particularly in relation to its expression and activity, may provide valuable insights into the molecular basis and therapeutic potential for epilepsy management. Although *Alg13* has been identified as a susceptibility gene for epilepsy in humans, the specific pathological mechanisms through which its mutations contribute to disease progression remain unclear.

Based on previous evidence, *Alg13* appears to play a role in regulating cortical interneuron development. The present research showed that deletion of *Alg13* markedly impaired postnatal cortical interneuron development and migration in mice (14). Based on previous evidence implicating *Alg13* in epilepsy susceptibility and cortical development, we hypothesized that *Alg13* plays a critical role in postnatal cortical interneuron migration and maturation. To test this hypothesis, we examined the spatiotemporal distribution of major GABAergic interneuron subtypes in the cerebral cortex of *Alg13* knockout mice at defined postnatal stages (P1, P7, and P30). In addition, we assessed interneuron migratory capacity using BrdU labeling and investigated transcriptional alterations associated with *Alg13* KO through bulk RNA sequencing and targeted gene validation. Together, these approaches were designed to elucidate the developmental role of *Alg13* in cortical interneuron development.

## 2 Materials and methods

### 2.1 Animals

*Alg13* knockout (KO) mice on a C57BL/6J background were generated via CRISPR-Cas9-mediated deletion of five nucleotides in exon 4 of the *Alg13* gene, as provided by Dr. Baoli Yu. Genotyping of *Alg13* KO mice and their wild-type (WT) littermates was performed as previously described (15). All animals were maintained under specific pathogen-free (SPF) conditions in individually ventilated cages (IVC) at Ningxia Medical University. Experimental procedures involving animals were reviewed and approved by the Institutional Animal Care and Use Committee (IACUC) of Ningxia Medical University (IACUC Animal Using Certificate No. IACUC-NYLAC-2023-143). All procedures adhered to institutional and ethical guidelines for animal care and use. All experiments were performed using male C57BL/6J mice. Mice were randomly assigned to experimental groups.

### 2.2 Immunofluorescence

Postnatal day (P) P7 and P30 mice were anesthetized by intraperitoneal injection of 1% sodium pentobarbital. Following anesthesia, mice were fixed by cardiac perfusion. Dissection was performed from the subxiphoid process until full exposure of the heart. A perfusion needle was inserted from the apex of the heart toward the aorta, and the right atrium was incised to allow blood replacement with saline through the perfusion system. After clear effluent was observed from the right atrial incision, perfusion was continued with 4% paraformaldehyde until whole-body rigidity was achieved, indicating successful fixation. The brain was then excised and post-fixed in 4% paraformaldehyde for 15 h at room temperature.

Following fixation, brains were trimmed with a thin blade and rinsed in tap water for 1 h. Samples were then dehydrated in a graded ethanol series (50, 70, 95, 100% I, 100% II, 100% III) for 10 min each and cleared sequentially in ethanol-xylene mixtures (ethanol 2:1 xylene, ethanol 1:1 xylene) and 100% xylene I–III for 10 min each. Tissues were infiltrated with molten paraffin I and II for 60 min, embedded using a paraffin embedding system, solidified, and stored at −20 °C before sectioning. Paraffin blocks were sectioned at 5 μm thickness using a rotary microtome (Leica, Germany). Sections were mounted on polylysine-coated slides and baked at 58 °C for approximately 1 h.

Slides were dewaxed and rehydrated through sequential immersion in xylene I and II, anhydrous ethanol I and II, 95, 80, and 70% ethanol, followed by purified water and phosphate-buffered saline (PBS) (xylene for 10 min, others for 5 min each). Antigen retrieval was conducted using Tris-EDTA buffer under boiling conditions for 10 min, followed by cooling and washing in PBS. Permeabilization was performed using 0.5% Triton X-100 (diluted in PBS) at 37 °C for 30 min. After removing residual solution, sections were incubated with 5% normal goat serum (ZLI-9022, ZSGB-BIO) for 1 h at room temperature to block nonspecific binding. Primary antibodies were applied overnight at 4 °C, followed by 1-h rewarming at room temperature the next day. Sections were washed three times in PBS for 5 min each, then incubated with biotinylated goat anti-rabbit IgG for 1 h at room temperature, followed by additional PBS washes. After staining, DAPI (G1012-10ML, Servicebio INC) was applied to counterstain nuclei. Sections were dried and imaged using a Leica fluorescence microscope (Leica Camera AG, Wetzlar, Germany). Positive immunofluorescent expression was identified by red or green fluorescence signals. For each paraffin block, at least six sections were analyzed, and 3 to 6 regions of interest were randomly selected per section. Image-Pro Plus 6.0 software was used to quantify the average optical density (IOD/area), and these values were used to evaluate protein expression levels. Each experimental group included three mice, with data combined from six sections per mouse (14).

### 2.3 Intraperitoneal injection of BrdU

Bromodeoxyuridine (BrdU; GC31002-100 mg, Servicebio INC) was dissolved in saline to a final concentration of 10 mg/mL. A single intraperitoneal injection of BrdU (50 mg/kg) was administered to mice at postnatal day 1 (P1). Brain tissues were collected on postnatal day 7 (P7) following saline and paraformaldehyde perfusion.

### 2.4 Transcriptome sequencing

Cortical tissues were collected from the cerebral cortex of male *Alg13* knockout and wild-type mice at postnatal days P1, P7, and P30. Total RNA was extracted using TRIzol reagent according to the manufacturer’s instructions. RNA concentration and purity were assessed using a NanoDrop 2000 spectrophotometer, and RNA integrity was evaluated using an Agilent 2100 Bioanalyzer. Only samples with an RNA integrity number (RIN) ≥7.0 were used for subsequent library construction. RNA libraries were prepared using the VAHTS Universal V6 RNA-seq Library Prep Kit following the manufacturer’s protocol. Sequencing was performed on an Illumina platform to generate paired-end reads. Raw sequencing data were subjected to quality control to remove adaptor sequences and low-quality reads. Clean reads were aligned to the reference mouse genome, and gene expression levels were quantified as fragments per kilobase of transcript per million mapped reads (FPKM). Differential expression analysis was conducted using standard bioinformatic pipelines, with adjusted *p* values used to determine statistical significance. Transcriptome sequencing analysis was conducted on cortical tissue samples collected from mice at postnatal days P1, P7, and P30 to examine the relationship between differential gene expression, neuronal development, and epilepsy susceptibility in *Alg13*-deficient mice. A total of 18 samples were sequenced, yielding 109.43 GB of clean data. The effective data volume per sample ranged from 5.48 to 7.0 GB, with Q30 base distributions between 92.6 and 93.72% and an average GC content of 48.00%. Alignment of sequencing reads with the reference genome demonstrated a mapping rate of 98.05 to 98.56%. Based on these alignments, expression analyses of protein-coding genes were performed. Transcriptome sequencing and bioinformatic analysis were performed by OE Biotech Co., Ltd. (Shanghai, China). All raw and processed RNA-seq data are publicly available through the GEO database.

### 2.5 RT-qPCR

Cortical tissues were isolated from the cerebral cortex of P7 mice. Total RNA was reverse-transcribed using the RevertAid First Strand cDNA Synthesis Kit (91334515, Thermo Scientific) in accordance with the manufacturer’s protocol. Quantitative polymerase chain reaction (RT-qPCR) was performed using the QuantiNova SYBR PCR Mix Kit (4993626, QIAGEN). *Gapdh* served as the internal reference gene, and relative mRNA expression levels were calculated using the Livak (2^–ΔΔCt^) method.

### 2.6 Statistical methods

All statistical analyses were performed using GraphPad Prism (version 10; GraphPad Software, San Diego, CA, United States). Data are presented as mean ± standard deviation (SD). Comparisons between two groups were performed using Student’s *t*-test, while multiple group comparisons were analyzed by one-way analysis of variance (ANOVA) followed by the least significant difference (LSD) *t*-test. A *p*-value <0.05 was considered statistically significant. Analyses were performed to evaluate differences in the spatiotemporal distribution of cortical interneurons between *Alg13* knockout (KO) and wild-type (WT) mice. For each experimental group, six male C57BL/6J mice were analyzed, and all experiments were independently repeated three times. Individual mice were considered as biological replicates for statistical analysis.

## 3 Results

Breeding and genotyping of *Alg13* KO mice were successfully achieved, ensuring the maintenance of homozygous *Alg13* KO lines (15). For statistical analysis using GraphPad, the mouse cerebral cortex was divided into 10 regions from superficial to deep layers. The first region corresponded to cortical layer I; the second and third regions represented layers II and III; the fourth and fifth represented layer IV; the sixth and seventh corresponded to layer V; and the eighth, ninth, and tenth represented layer VI. The number of interneurons in each region was quantified by a blinded researcher who was unaware of the genotypes.

### 3.1 Spatial association between *Alg13* expression and major interneuron subtypes in the postnatal mouse cerebral cortex

**Figure 1:**
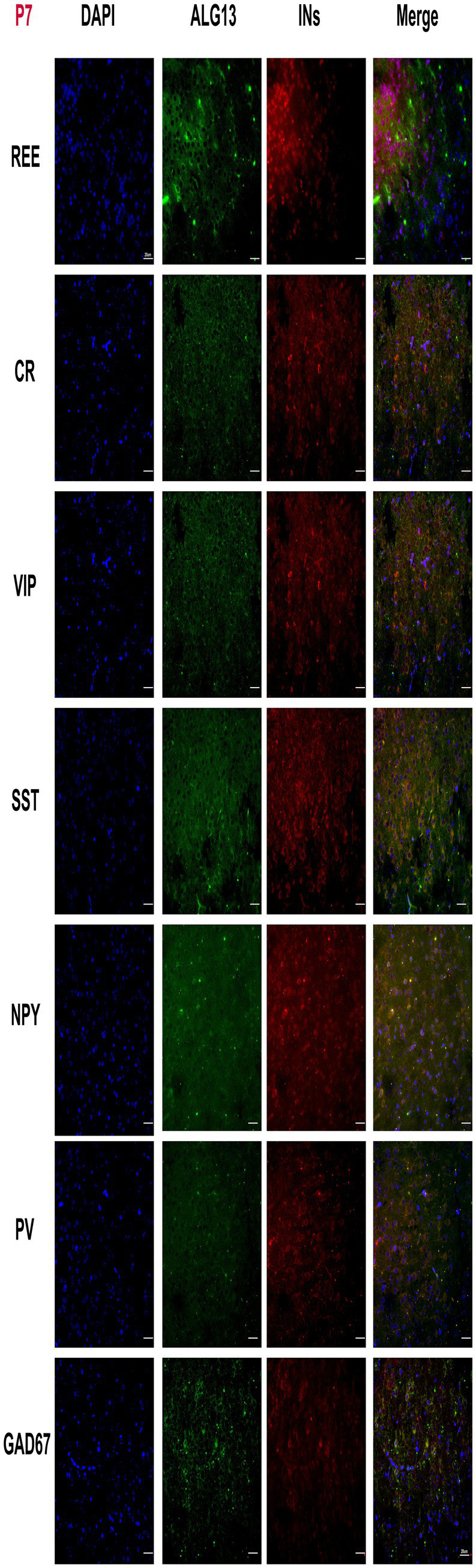
Fluorescent microscopy panel showing the cortical cortex of WT mice stained with DAPI (blue), ALG13 (green), and interneuron markers (red) across seven rows labeled REE, CR, VIP, SST, NPY, PV, and GAD67. Each row displays four columns for DAPI, ALG13, interneurons, and merged images, highlighting nuclear, ALG13, and interneuronal distribution and colocalization in each group at P7. Scale bars indicate 20 micrometers.

**Figure 2:**
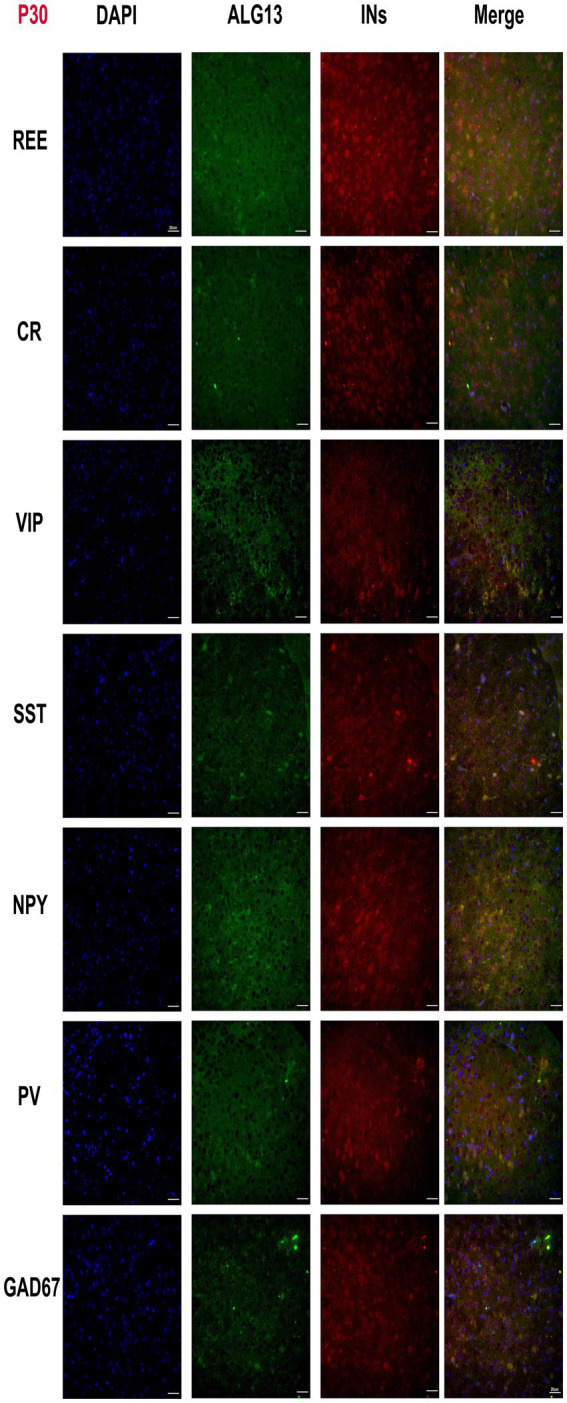
Fluorescent microscopy panel showing the cortical cortex of WT mice stained with DAPI (blue), ALG13 (green) and interneuron markers (red) across seven rows labeled REE, CR, VIP, SST, NPY, PV and GAD67 at P30. Each row displays four columns for interneurons, DAPI, ALG13 and merged images, highlighting nuclear, ALG13 and interneuronal distribution and colocalization in each group at P30. Scale bars indicate 20 micrometers.

Neuronal subtypes perform distinct roles in the central nervous system, and their functional properties vary significantly. Previous research has shown that *Alg13 KO* causes structural and functional abnormalities in the mouse cerebral cortex and altered interneuron number and spatial distribution. Immunofluorescence analysis at postnatal days P7 and P30 revealed that *Alg13* expression was spatially associated with multiple GABAergic interneuron subtypes, including PV-, SST-, VIP-, NPY-, CR-, and REE-positive cells in the cerebral cortex (Figures 1, 2).

These observations indicate that *Alg13* is broadly expressed in cortical regions populated by diverse interneuron subtypes. However, these data represent qualitative spatial overlap rather than definitive molecular co-localization, and do not establish interneuron lineage specification or fate determination. The analysis was intended to provide a descriptive overview of *Alg13* expression patterns during postnatal cortical development.

### 3.2 *Alg13* deletion reducing GAD67 expression and altering interneuron distribution during early postnatal development

**Figure 3:**
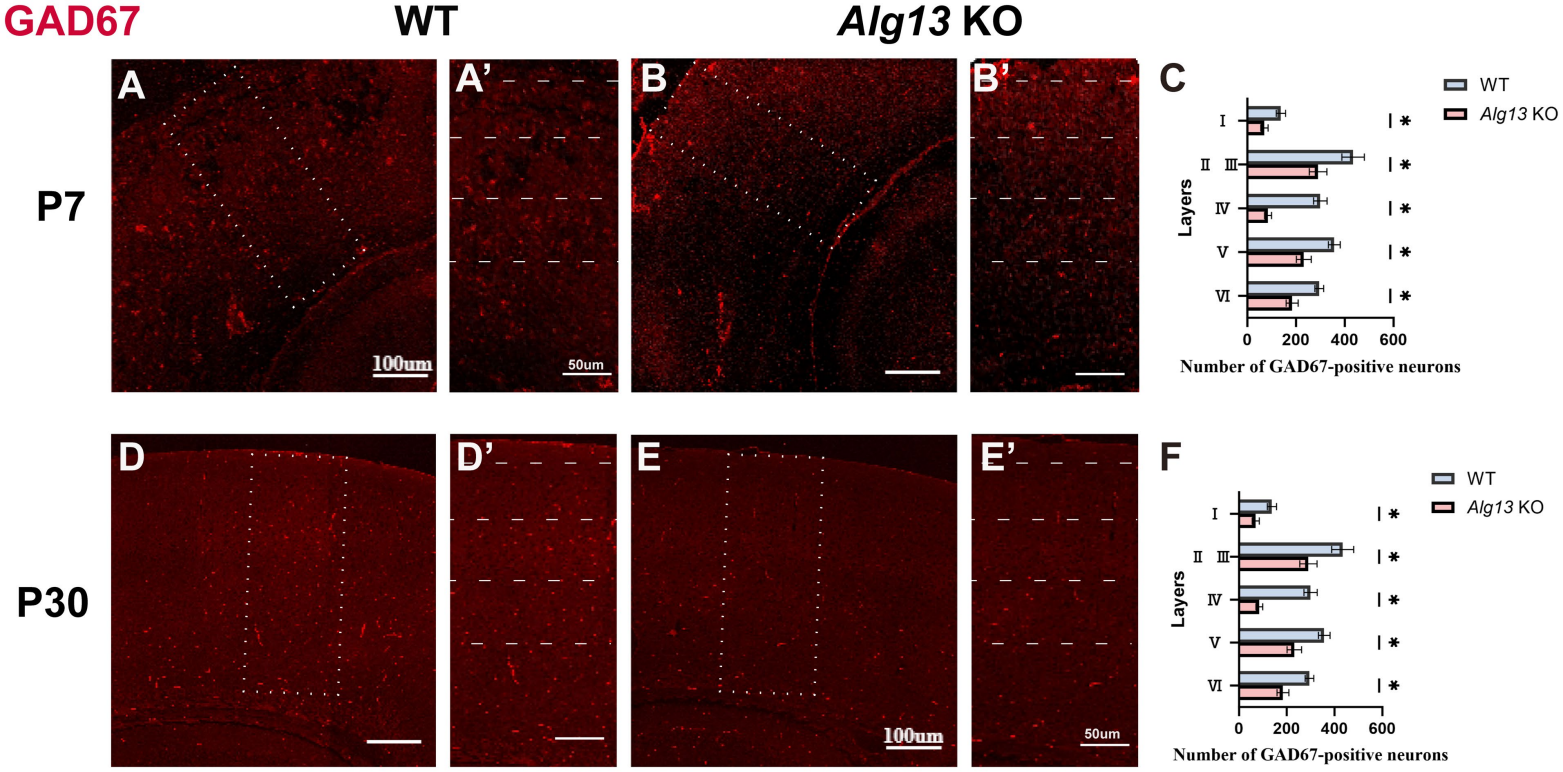
Immunofluorescence images and bar graphs comparing GAD67-positive interneurons in cortical cortex of WT and *Alg13* KO mice at P7 and P30. **(A, B, D, E)** The immunofluorescent images showing GAD67-positive interneurons in cortical cortex of WT and *Alg13* KO mice at P7 and P30. Scale bars indicate 100μm. **(A′, B′, D′, E′)** The high magnification views corresponded to the area marked by a dashed rectangle in A, B, D, E. Sections display labeled neuron distributions across cortical layers I to VI. Scale bars indicate 50μm. **(C,F)** Quantification graphs comparing the laminar distribution and overall counts of cumulative interneurons in WT and *Alg13* KO mice at P7 and P30. (***p < 0.001, **p < 0.01, and *p < 0.05).

Previous findings have indicated that *Alg13* KO in mice affects interneuron development and tangential migration (10). Glutamate decarboxylase 67 (GAD67) was used as a marker to assess interneuron quantity and distribution. At postnatal day 7, a marked decrease was observed in the number of interneurons in layer IV and in the laminar distribution across other cortical layers in *Alg13* KO mice compared with WT controls. By P30, the total number of interneurons remained reduced, although the laminar distribution demonstrated no significant differences between groups. These findings suggest partial recovery of laminar organization during postnatal development; however, the total interneuron count did not recover within the same period, emphasizing the critical role of *Alg13* in postnatal interneuron maturation. At postnatal day 7 (P7), immunofluorescence analysis revealed a significant reduction in GAD67-positive interneurons across all cortical layers in *Alg13* KO mice compared with WT controls. This widespread decrease suggests that *Alg13* deletion leads to a global impairment of GABAergic interneuron maturation and/or tangential migration during early postnatal development, rather than a defect restricted to specific cortical layers (Figures 3A–C), whereas by P30, no significant laminar differences were detected (Figures 3D–F). By postnatal day 30 (P30), no significant differences in the laminar distribution of GAD67-positive interneurons were observed between WT and *Alg13* KO mice. This finding suggests that laminar positioning of interneurons may undergo partial normalization during adolescent development. However, despite the apparent recovery of laminar organization, the overall number of GAD67-positive interneurons remained lower in *Alg13* KO mice, indicating that early postnatal deficits were not fully compensated. This gradual restoration of laminar organization suggests that *Alg13* deletion disrupts early postnatal interneuron development, with partial compensatory recovery occurring during maturation. Collectively, these results indicate that *Alg13* deletion causes a pronounced reduction in GABAergic interneuron maturation during early postnatal development, with partial restoration of laminar distribution but persistent deficits in interneuron number at later stages.

### 3.3 Different subtypes of interneurons exhibiting the abnormal density and distribution in the cortex after *Alg13* ablation

Cortical interneurons represent a highly heterogeneous neuronal population characterized by diverse morphologies, connectivity patterns, biochemical properties, and physiological functions (16). Their laminar distribution and subtype composition are essential for maintaining cortical network stability and neuronal signaling (17). Immunofluorescence analysis was performed to compare PV-, SST-, calretinin (CR)-, REE-, vasoactive intestinal peptide (VIP)-, and NPY-positive interneurons between WT and *Alg13* KO mice.

PV- and SST-positive interneurons, primarily derived from the medial ganglionic eminence (MGE) and subpallial regions, are major inhibitory neuron types typically distributed in cortical layers V and VI (18). VIP- and NPY-positive interneurons originate mainly from the caudal ganglionic eminence (CGE), comprising approximately 50% of CGE-derived interneurons, and are predominantly located in superficial cortical layers (19). CR-positive interneurons arise from both the CGE and MGE and are mainly distributed in superficial cortical layers, whereas REE-positive interneurons are broadly distributed across multiple cortical layers (20, 21).

**Figure 4:**
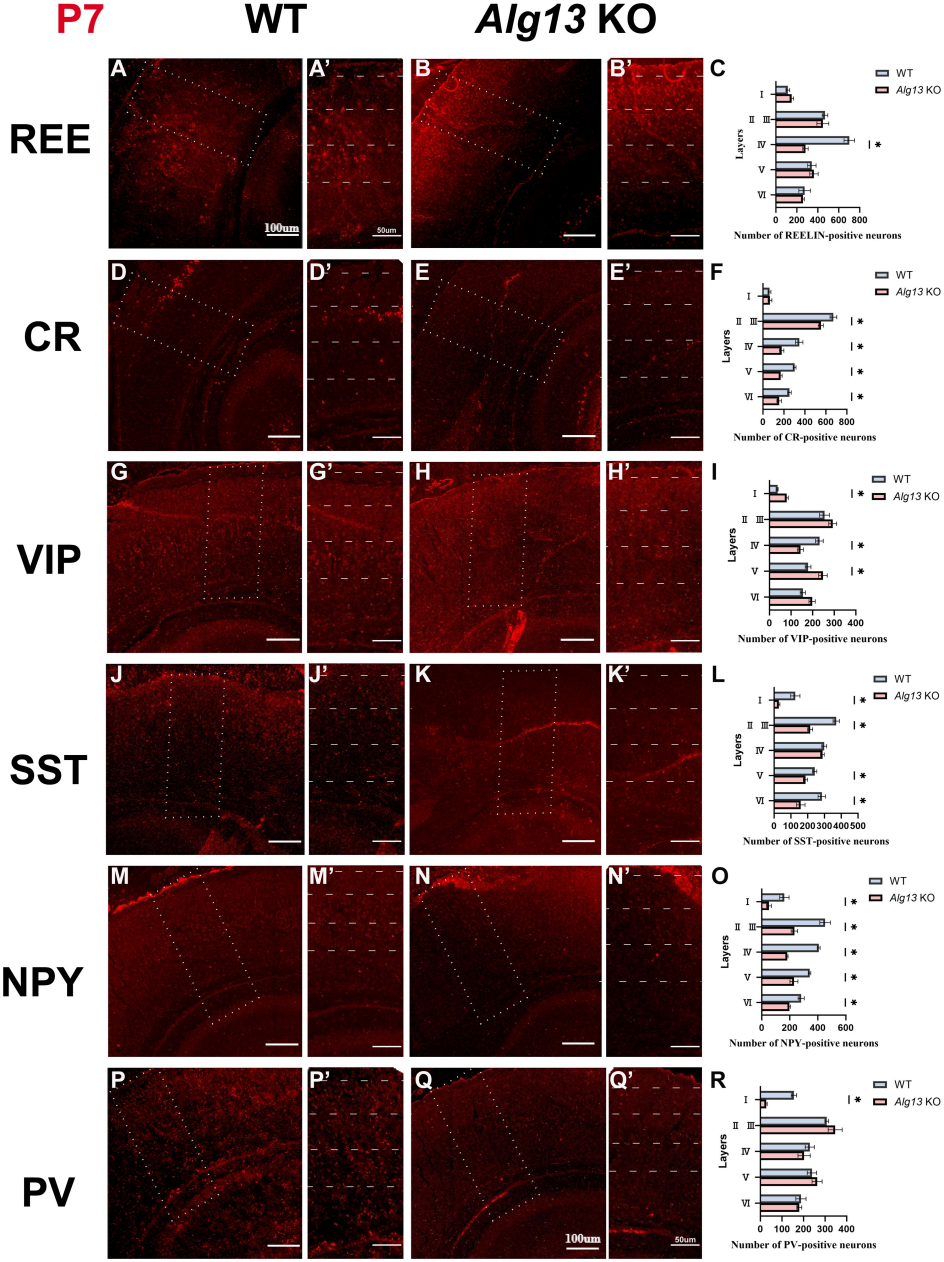
Immunofluorescence images and bar graphs comparing different interneuronal populations (REE, CR, VIP, SST, NPY, PV) in cortical cortex between WT and *Alg13* KO mice at P7. **(A,B,D,E,G,H,J,K,M,N,P,Q)** The immunofluorescent images showing different interneurons in cortical cortex of WT and *Alg13* KO mice at P7. Scale bars indicate 100μm. **(A′,B′,D′,E′,G′,H′,J′,K′,M′,N′,P′,Q′)** The high magnification views corresponded to the area marked by a dashed rectangle in A, B, D, E, G, H, J, K, M, N, P, Q. Sections display labeled neuron distributions across cortical layers I to VI. Scale bars indicate 50μm. **(C,F,I,L,O,R)** Quantification graphs comparing the laminar distribution and overall counts of different interneurons in WT and *Alg13* KO mice at P7. (***p < 0.001, **p < 0.01, and *p < 0.05).

At P7, immunofluorescence analysis revealed that *Alg13* KO mice exhibited significant reductions in SST-, PV-, and VIP-positive neurons in superficial cortical layers. PV- and SST-positive interneurons, which are normally concentrated in deeper layers, were instead observed more frequently in superficial regions, indicating an “inside-out” laminar distribution that could disrupt inhibitory signaling and cortical network stability (22). CR- and NPY-positive neurons demonstrated widespread reductions across cortical regions, and REE-positive neurons were markedly decreased in layer IV at P7 (Figures 4A–R).

**Figure 5:**
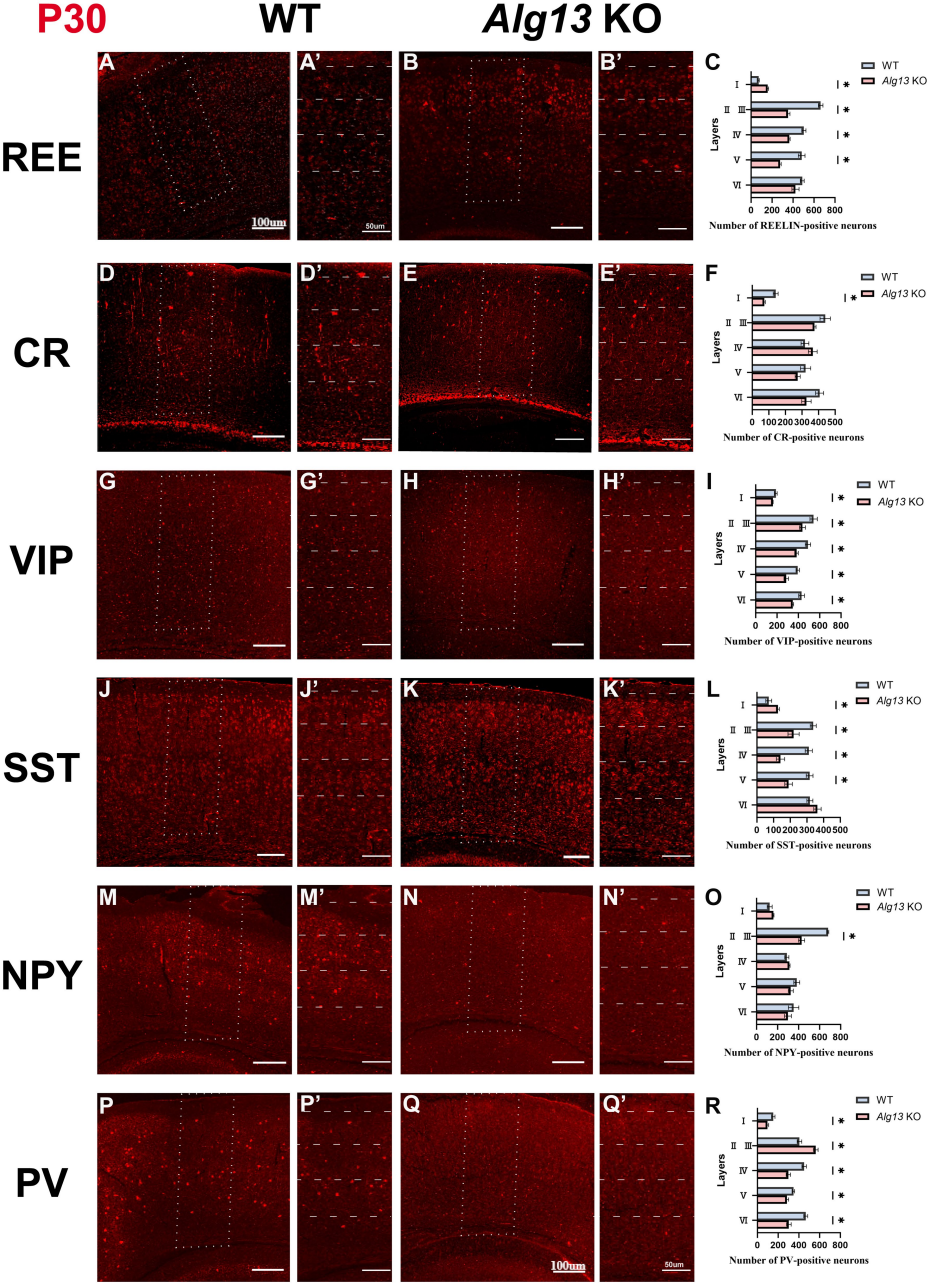
Immunofluorescence images and bar graphs comparing different interneuronal populations (REE, CR, VIP, SST, NPY, PV) in cortical cortex between WT and *Alg13* KO mice at P30. **(A,B,D,E,G,H,J,K,M,N,P,Q)** The immunofluorescent images showing different interneurons in cortical cortex of WT and *Alg13* KO mice at P30. Scale bars indicate 100μm. **(A′,B′,D′,E′,G′,H′,J′,K′,M′,N′,P′,Q′)** The high magnification views corresponded to the area marked by a dashed rectangle in A, B, D, E, G, H, J, K, M, N, P, Q. Sections display labeled neuron distributions across cortical layers I to VI. Scale bars indicate 50μm. **(C,F,I,L,O,R)** Quantification graphs comparing the laminar distribution and overall counts of different interneurons in WT and *Alg13* KO mice at P30. (***p < 0.001, **p < 0.01, and *p < 0.05).

At P30, PV-positive interneurons demonstrated decreased distribution across most cortical layers in *Alg13* KO mice, except for layers II and III, where their numbers and density slightly increased despite an overall reduction. SST- and VIP-positive neurons demonstrated significant reductions across all layers compared with WT controls. The number of CR- and REE-positive neurons in layer I was also significantly reduced in *Alg13* KO mice. Additionally, NPY-positive neurons were markedly decreased in layers II and III, with an overall reduction in total NPY interneuron numbers. These findings suggest that *Alg13* deletion caused persistent abnormalities in interneuron subtype density and laminar distribution during postnatal cortical development (Figures 5A–R).

### 3.4 Impaired migration of interneurons after *Alg13* deletion

**Figure 6:**
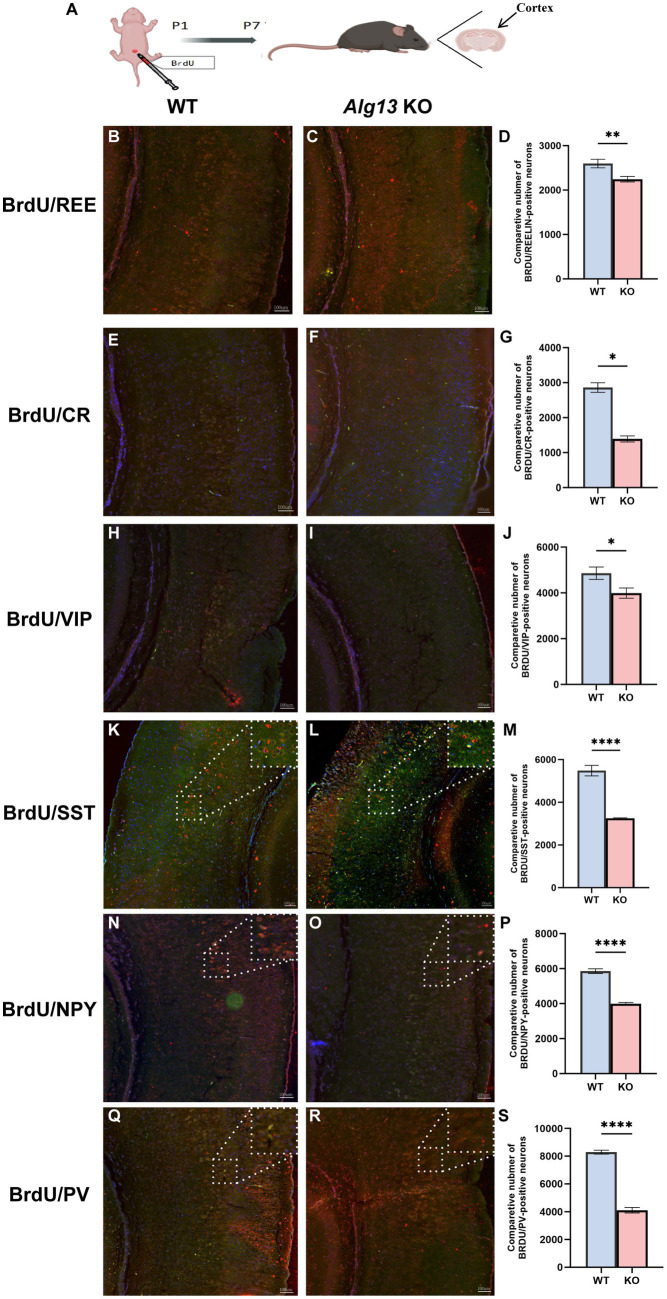
The co-localization of BrdU with specific neuronal subtypes in the cerebral cortex of WT and Alg13 knockout mice at P7. **(A)** Diagram shows a schematic of BrdU injection in WT and *Alg13* KO mice at P1 with cortical analysis at P7. **(B,C,E,F,H,I,K,L,N,O,Q,R)** Immunofluorescence images comparing interneurons in WT mice and *Alg13* KO mice, with CR, VIP, SST, NPY, PV-positive interneurons co-expressed with BrdU+ at P7. Scale bars indicate 100μm. **(D,G,J,M,P,S)** Quantification bar graphs for each marker reveal significant reductions in BrdU-positive interneurons in KO mice compared to WT at P7. (***p < 0.001, **p < 0.01, and *p < 0.05).

Interneurons migrate to the dorsal cortex primarily via two routes: the superficial stream in the marginal zone and the deep stream in the subventricular zone (SVZ) or intermediate zone (IZ). Tangential migration is a key developmental process regulated by chemotaxis, motility, transcription factors, and neurotransmitters (23). To evaluate interneuron migration, BrdU was administered intraperitoneally at P1, and brain tissues were collected at P7. Neuronal populations in the cerebral cortex include both migrating neurons and those generated by *in situ* proliferation (Figure 6A). Because BrdU labeling inhibits local proliferation, it allows distinction between neurons arising through migration and proliferation.

The co-localization of BrdU with specific neuronal subtypes in the cerebral cortex was used to assess migratory capacity. Immunofluorescence results demonstrated that *Alg13* deletion significantly reduced the number of BrdU-labeled interneurons reaching the cortex (Figures 6B–S). Reductions were most pronounced for SST-, NPY-, and PV-positive subtypes (*p* < 0.0001) (Figures 6K–S). Furthermore, numerous ectopic neurons were observed in the deeper cortical layers of *Alg13* KO mice, indicating disrupted migratory patterns. These findings suggest that *Alg13* deletion impairs postnatal cortical interneuron migration, potentially contributing to increased seizure susceptibility in affected mice.

### 3.5 Transcriptome sequencing results of the WT group and the *Alg13* KO group

**Figure 7:**
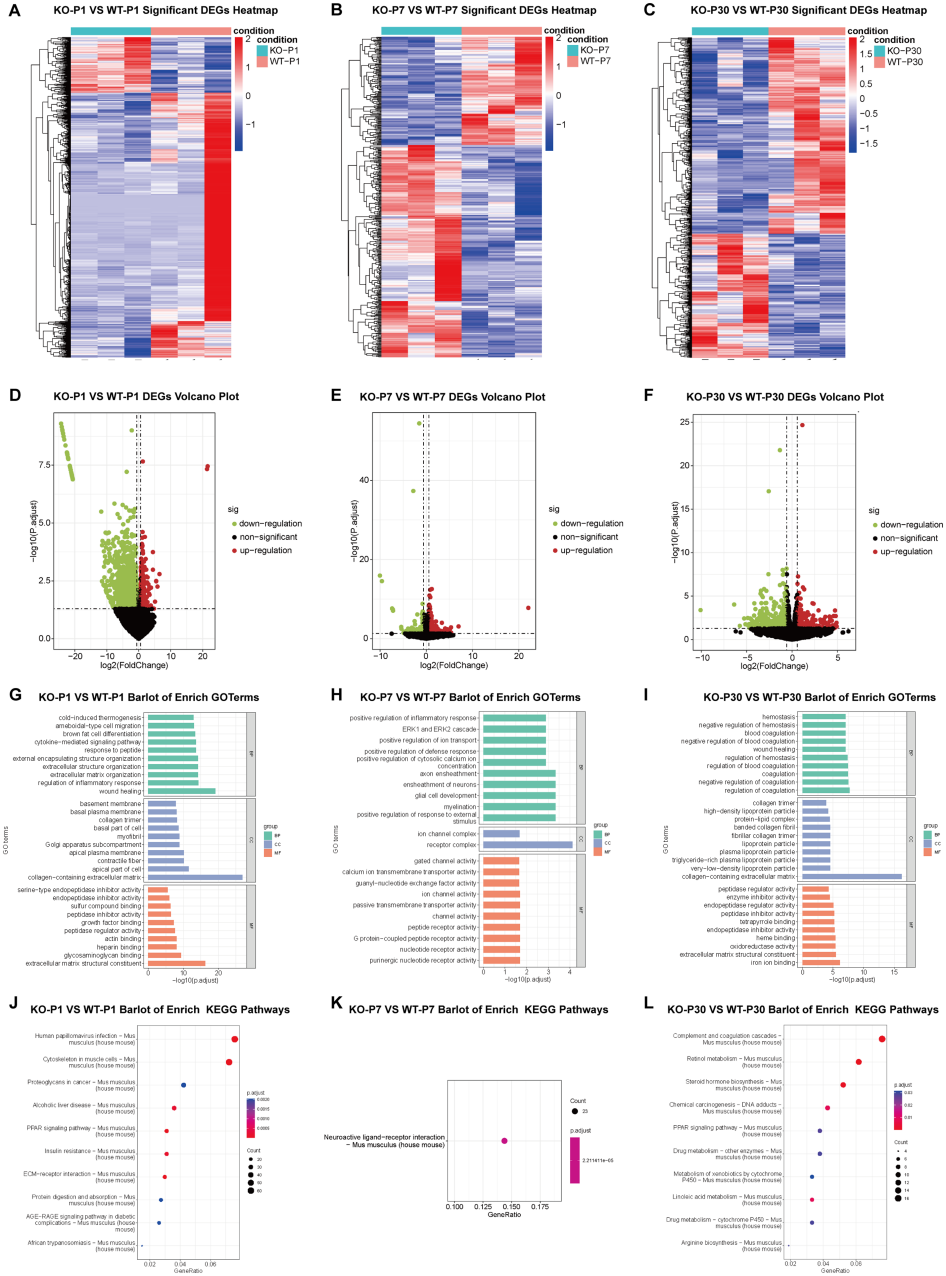
DEG identification and multivariate statistical analysis of WT and *Alg13* KO mice in transcriptome studies at three time points: P1, P7 and P30. **(A–C)** Clustering heatmaps illustrate the clustering and expression patterns of significant differentially expressed genes. **(D–F)** Volcano plots display the up- and down-regulated DEGs based on log2 fold change. **(G–I)** Enriched Gene Ontology (GO) terms highlight biological processes associated with neuronal development, migration, and synaptic organization among differentially expressed genes. **(J–L)** Enriched KEGG pathways shows counts, gene ratios, and adjusted *p*-values.

Differential expression screening identified 1,403, 231, and 358 differentially expressed genes at the respective time points. Expression levels at each developmental stage were analyzed, heatmap analysis revealed stage-specific transcriptional signatures following *Alg13* deletion, with distinct gene clusters associated with neuronal differentiation at P1, interneuron migration and maturation at P7, and synaptic organization at P30. These expression patterns indicate that *Alg13* deletion leads to coordinated dysregulation of gene networks governing interneuron migration and maturation rather than isolated gene-specific effects (Figures 7A–C). Volcano plots similarly illustrated these patterns, with red indicating upregulation and green representing downregulation at each time point (Figures 7D–F). Gene Ontology (GO) and Kyoto Encyclopedia of Genes and Genomes (KEGG) pathway enrichment analyses were conducted to characterize significant biological processes (BP), cellular components (CC), and molecular functions (MF) associated with these genes. GO enrichment analysis revealed that differentially expressed genes at P7 were predominantly associated with neuronal migration, axon guidance, and regulation of GABAergic signaling, consistent with the observed defects in interneuron positioning and GAD67 expression. In contrast, GO terms enriched at P30 were mainly related to synaptic organization and neuroimmune processes, suggesting long-term effects of early transcriptional dysregulation on cortical circuit maturation. At P1, enriched GO terms were primarily related to early neuronal differentiation and cytoskeletal organization, indicating that *Alg13* KO may influence initial postnatal developmental programs. At P7, a critical window for interneuron migration, GO terms associated with neuronal migration, neurite outgrowth, and inhibitory synapse development were significantly enriched, providing a molecular basis for the pronounced interneuron distribution and maturation defects observed at this stage. At P30, enriched GO terms shifted toward synaptic and immune-related processes, suggesting that early developmental perturbations induced by *Alg13* loss may lead to persistent alterations in cortical circuit regulation rather than ongoing defects in neuronal positioning (Figures 7G–L). Differential expression analysis revealed that *Alg13* KO induces stage-specific transcriptional alterations associated with neuronal development and interneuron maturation. At P1, differentially expressed genes were predominantly enriched in GO terms related to cell cycle regulation, early neuronal differentiation, and cytoskeletal organization, suggesting that *Alg13* loss may affect early postnatal neuronal developmental programs. At P7, a critical period for interneuron migration and laminar positioning, differentially expressed genes were significantly enriched in biological processes associated with neuronal migration, axon guidance, synaptic assembly, and regulation of GABAergic signaling, consistent with the pronounced defects in interneuron distribution and GAD67 expression observed at this stage. By P30, transcriptional changes were primarily associated with synaptic organization, neuronal projection development, and neuroimmune-related pathways, indicating persistent but altered regulatory programs during adolescent cortical maturation despite partial recovery of laminar organization.

**Figure 8:**
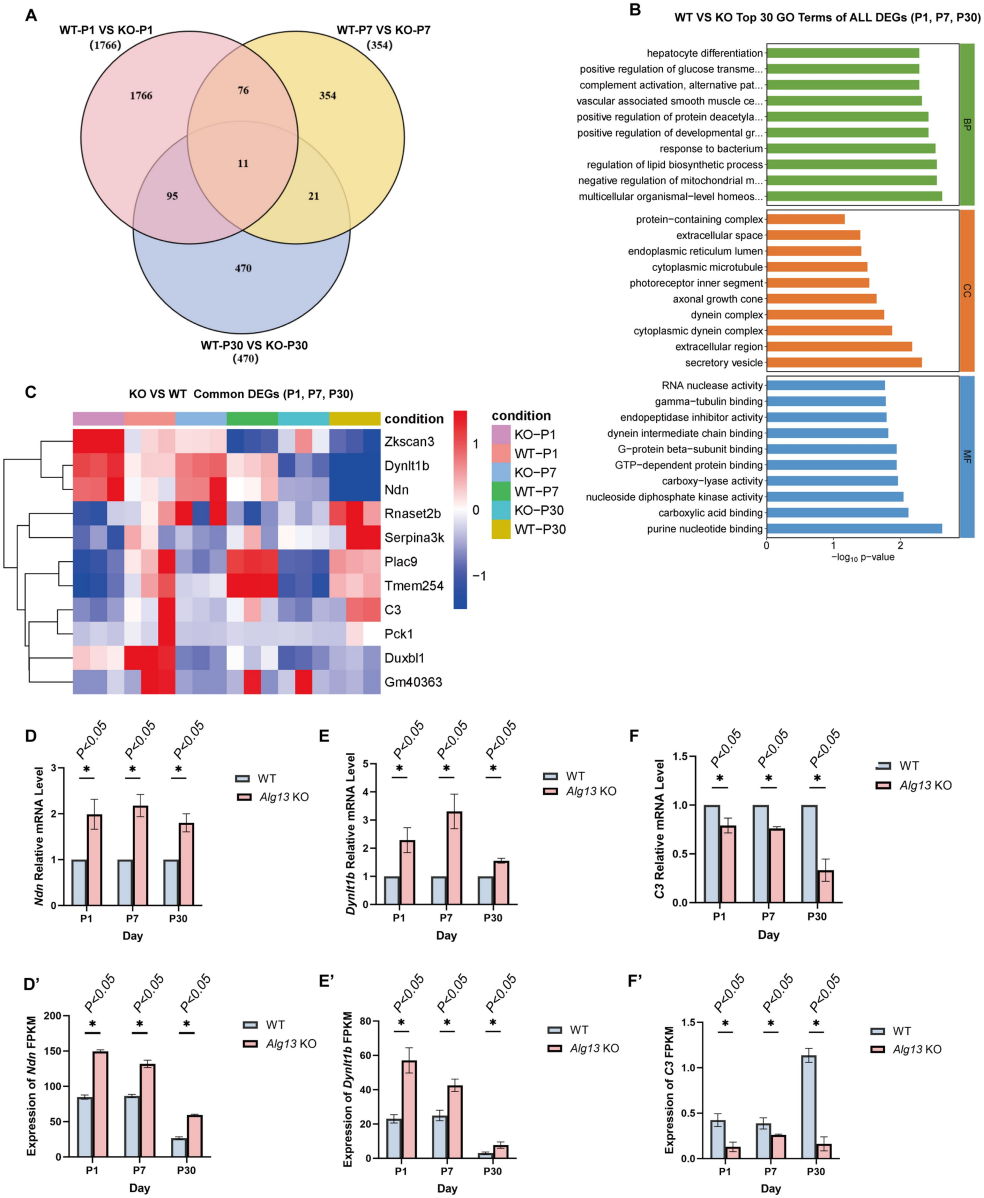
Screen and validation of shared differentially expressed genes in WT and *Alg13* KO mice across three postnatal stages. **(A)** Venn diagram shows shared and unique differentially expressed genes between WT and *Alg13* KO groups at P1, P 7, and P 30. **(B)** Bar graphs display top gene ontology terms for biological process, cellular component, and molecular function. **(C)** Heatmap depicts expression patterns of common differentially expressed genes across conditions and time points. **(D–F)** Statistical plots show the RT-qPCR expression levels for *Ndn*, *Dynlt1b*, and C3. **(D′–F′)**Statistical plots show the FPKM expression values for *Ndn*, *Dynlt1b*, and *C3*. (*p < 0.05).

Venn analysis identified 11 shared differentially expressed genes across the three developmental stages. These 11 genes represent commonly dysregulated candidate targets associated with *Alg13* KO across postnatal developmental stages. Gene set enrichment analysis using the Molecular Signatures Database (MSigDB) identified three genes associated with interneuron development: *Ndn*, *Dynlt1b*, and *C3* (Figures 8A–C). *Ndn* encodes necdin, a member of the MAGE family of neurodevelopmental proteins that act upstream of several neurogenic processes (24). Previous evidence has shown that *Ndn* promotes the differentiation of GABAergic neurons during forebrain development (25). Embryos lacking *Ndn* demonstrated a significant reduction in the number of cells migrating from the MGE to the neocortex and exhibited heightened sensitivity to pentylenetetrazole-induced seizures. Additionally, *Ndn* may be involved in maintaining intracellular calcium homeostasis in neurons (26).

*Dynlt1b* encodes a dynamin light chain of the Tctex-1B type, which contributes to neurite initiation, germination, and axon formation (27). Overexpression of Tctex-1 has been associated with inhibitory effects on these processes. *Dynlt1b* also interacts with G-protein β subunits and GTP-dependent protein complexes, playing roles in mitotic spindle orientation, negative regulation of neurogenesis, and modulation of G-protein-coupled receptor signaling (28). It acts upstream of the ERK1/2 signaling cascade and promotes neuronal projection development, regulating several stages of neuronal maturation (29).

*C3* encodes complement component 3, which occupies a central role in activating the complement cascade (30). It participates in both the classical and alternative complement pathways through cleavage by *C3* convertase enzymes (31). Upon activation, *C3*b covalently binds to cell surface carbohydrates or immune complexes via its reactive thioester group (32).

Validation of transcriptomic findings was performed using RT-qPCR. The expression trends of *Ndn*, *Dynlt1b*, and *C3* obtained by RT-qPCR were consistent with their fragments per kilobase of transcript per million mapped reads (FPKM) values. Compared with the WT group, *Alg13* KO mice demonstrated significant upregulation of *Ndn* and *Dynlt1b* and downregulation of *C3* (*p* < 0.05) (Figures 8D–Fʹ). These findings suggest that *Alg13* deletion may alter neuronal morphology and function by regulating the expression of genes involved in neurodevelopment.

## 4 Discussion

Findings from prior research indicate that deficiency of the *Alg13* gene increases epilepsy susceptibility and seizure severity in mice. The development of higher cognitive functions in the cerebral cortex depends on the coordinated activity of two major classes of neurons, excitatory and inhibitory (14). Interneurons have been reported to primarily originate from neural epithelial stem cells in the ventricular zone and from intermediate progenitor cells in the SVZ of the ventral telencephalon’s subpallium (33, 34). Following expression of *DLX2*, *NKX2-1*, and *LHX6*, these progenitor cells differentiate into interneuron subgroups and subsequently migrate to specific brain regions (35). The marked reduction of GAD67 expression across all cortical layers at P7 suggests that *Alg13* plays a critical role during early postnatal stages when interneuron migration and maturation are still actively ongoing. Loss of *Alg13* may delay interneuron maturation, reduce GABA synthesis capacity, or impair migration efficiency, resulting in a widespread decrease in GAD67-positive cells. The absence of significant laminar differences at P30 may reflect developmental compensation, circuit stabilization, or delayed maturation of surviving interneurons during adolescence. Nevertheless, the persistent reduction in overall interneuron number indicates that early postnatal deficits are not fully reversible, highlighting the importance of *Alg13* during a critical developmental window. Interneurons can be classified according to their molecular characteristics and region of origin. The MGE predominantly produces PV+ and SST+ interneurons, accounting for approximately 40 and 18% of total interneurons, respectively (36, 37). The CGE primarily generates VIP+, NPY+, CR+, and REE-positive (REE+) interneurons, with CR+ and VIP+ subtypes together comprising about 25% of cortical interneurons (38). The preoptic area (POA) gives rise to additional subtypes, including PV+, SST+, and REE+ interneurons (39). In the present study, both the number and distribution of interneurons were significantly altered in *Alg13* KO mice. Aberrations in interneuron development and maturation have been associated with various neuropsychiatric disorders, including epilepsy, Rett syndrome (40), West syndrome, autism spectrum disorder, and Alzheimer’s disease (41–43). Therefore, these findings suggest new insights into the regulatory mechanisms of postnatal cortical interneuron development and provide a better understanding of the pathogenesis of *Alg13*-associated epilepsy.

The mammalian cerebral cortex is characterized by a six-layered laminar architecture with distinct morphological and functional organization (44). During neocortical development, neurons are generated from precursor cells located in the ventricular zone, undergo transformation from a multipolar to a bipolar morphology, and migrate radially along glial fibers to the cortical surface, where they integrate into neural circuits (45). Neuronal migration is essential for proper cortical structure formation. In the current study, BrdU administration one day after birth revealed that neuronal migration was markedly impaired by postnatal day 7 (P7). Previous data indicated that *Alg13* KO mice demonstrated increased susceptibility to epilepsy, potentially resulting from disrupted interneuron development and migration, a finding partially confirmed in this investigation. Quantitative analyses revealed that the numbers of PV+, SST+, CR+, VIP+, NPY+, and REE+ interneurons in the cerebral cortex were significantly reduced in *Alg13* KO mice, indicating that *Alg13* KO inhibited migration of these neuronal subtypes. Notably, the suppression of REE+, CR+, and VIP+ interneurons appeared particularly pronounced, which may be due to limited effects of postnatal BrdU on their migration or to selective vulnerability of certain GABAergic interneuron subtypes to *Alg13 KO*. This selective vulnerability suggests that specific neuronal subtypes may be more susceptible to developmental disruption under adverse conditions, including maternal inflammation and other environmental stressors.

Transcriptomic analysis revealed altered expression of genes associated with interneuron development in *Alg13* KO compared with WT mice, including *Dynlt1b*, *Ndn*, and *C3*. The *Dynlt1b* gene encodes the dynamin light chain Tctex-1B-type. Tctex-1 proteins promote neurite initiation, axon formation, and outgrowth, whereas overexpression inhibits these processes, which may explain the impaired cortical interneuron development observed in *Alg13* KO mice (46). Expression quantitative trait loci analyses indicated that epilepsy-related single nucleotide polymorphisms may regulate expression of *PTPRO* and *GADD45A* in human brain tissue, potentially accounting for the increased epilepsy susceptibility associated with *Alg13* KO (26). The *Ndn* gene encodes the necdin protein, which is critical for the development of serotonergic and GABAergic neurons and for central respiratory regulation. Necdin and *Bmi1* function antagonistically in regulating proliferation of embryonic neural precursor cells (NPCs) in the developing neocortex. Necdin may suppress transmigration in multiple cellular environments by inhibiting *Bmi1* and exert antimitotic effects by repressing *Cdk1* transcription (47).

In addition, upregulation of the complement component *C3* was observed in *Alg13* KO mice. The *C3* factor plays a central role in the complement cascade, mediating both the classical and alternative pathways (48, 49). Increased expression of *C3* has been implicated in neuroinflammatory responses and neuronal injury. *C3*-deficient mice exhibit reduced neuroinflammation, and elevated levels of *C3*b-rich plasma exosomes have been proposed as potential biomarkers for cerebrovascular disease (22, 50). The complement system is essential for glial-mediated physiological and pathological processes in neuroimmune disorders. Prior research demonstrated that *C1q* and *C3* transcript and protein levels were elevated in both individuals with epilepsy and corresponding animal models. Consistent with these findings, *Alg13* KO mice exhibited higher epilepsy incidence and susceptibility compared to controls, possibly linked to increased *C3* expression (51). Dysregulation of *C3* may therefore contribute directly to neuronal damage and cell death observed in epilepsy. The transcriptomic alterations observed across P1, P7, and P30 further support a developmental stage–dependent role of *Alg13* in cortical interneuron maturation. The enrichment of migration- and differentiation-related GO terms at P7 aligns closely with the observed deficits in interneuron positioning and GAD67 expression, suggesting that transcriptional dysregulation during this critical postnatal window may underlie the structural and cellular phenotypes observed in *Alg13* KO mice. Notably, genes such as *Ndn* and *Dynlt1b*, which are implicated in neuronal differentiation, cytoskeletal dynamics, and intracellular transport, were consistently dysregulated, providing a potential molecular link between *Alg13* KO and impaired interneuron development. In contrast, later-stage transcriptional changes at P30 were more closely associated with synaptic and neuroimmune processes, indicating that early developmental perturbations may have long-lasting effects on cortical circuit maturation rather than being fully compensated.

Overall, the experimental results confirmed that *Alg13* KO disrupted interneuron development in postnatal mice, indicating that *Alg13* may influence GABAergic neuron maturation and migration, thereby increasing seizure susceptibility and severity. However, a limitation of this study is that the analyses were restricted to postnatal stages, leaving the embryonic role of *Alg13* during neurogenesis unexamined. Future investigations should elucidate *Alg13* function throughout the full developmental timeline of GABAergic neurons to provide mechanistic insight and potential therapeutic targets for *Alg13*-associated refractory epilepsy.

Additionally, all experiments were performed in male mice, and electrophysiological or behavioral assessments were not conducted to validate the epileptic phenotype. To strengthen the findings, future research should include patch-clamp recordings of identified interneuron subtypes, *in vivo* epilepsy susceptibility assays, and conditional *Alg13* deletion at various developmental stages to determine its temporal role in cortical interneuron maturation. It should be noted that the present study focused exclusively on postnatal stages (P1, P7, and P30), which represent key developmental windows for interneuron migration, laminar positioning, and cortical circuit maturation. The role of *Alg13* during embryonic neurogenesis and in fully mature adult brains was not addressed and warrants further investigation in future studies. A limitation of this study is that classical interneuron lineage and migration markers, such as DLX, NKX2.1, LHX6, and DCX, were not systematically examined in combination with *Alg13* expression. In addition, quantitative co-localization analyses were not performed. Future studies incorporating embryonic and adult stages with multi-level validation will be necessary to fully elucidate the mechanistic role of Alg13 in neuronal development.

## Data Availability

The original contributions presented in the study are included in the article/supplementary material, further inquiries can be directed to the corresponding authors.

## Glossary

GABA: γ-Aminobutyric acid
MGE: Medial ganglionic eminence
CGE: Caudal ganglionic eminence
*Alg13*: Asparagine-linked glycosylation 13 homolog
*ALG*1: Asparagine-linked glycosylation 1 homolog
*ALG*3: Asparagine-linked glycosylation 3 homolog
*ALG*6: Asparagine-linked glycosylation 6 homolog
KO: Knockout
CRISPR-Cas9: Clustered regularly interspaced short palindromic repeats
SPF: Specific pathogen free
IVC: Individually ventilated cages
BrdU: 5-Bromo-2′-deoxyuridine
RT-qPCR: Reverse transcription quantitative polymerase chain reaction
RNA: Ribonucleic acid
μL: Microlitre
rpm: Revolutions per minute
cDNA: Complementary deoxyribonucleic acid
LSD *t*-test: Least significant difference *t*-test
WT: Wild type
PV: Parvalbumin interneuron
SST: Somatostatin interneuron
VIP: Vasoactive intestinal peptide interneuron
NPY: Neuropeptide Y interneuron
REE: REE-expressing interneuron
CR: Calretinin interneuron
GAD67: Glutamic acid decarboxylase 67 interneuron
ASCL1: Achaete-scute family BHLH transcription factor 1
NOTCH: Neurogenic locus notch homolog protein
DLX2: Distal-less homeobox 2
NKX2-1: NK2 homeobox 1
LHX6: LIM homeobox 6
P: Postnatal day
GO: Gene Ontology
KEGG: Kyoto Encyclopedia of Genes and Genomes
BP: Biological process
CC: Cellular component
MF: Molecular function
VENN: Venn diagram
MSigDB: Molecular Signatures Database
*Ndn*: Necdin
*Dynlt1b*: Dynein light chain Tctex-type 1B
*C3*: Complement component 3
ERK1/ERK2: Extracellular signal-regulated kinase 1/2
CBP: Classical complement pathway
SBP: Alternative complement pathway
ASD: Autism spectrum disorder
BPD: Bipolar affective disorder
SVZ: Subventricular zone
VZ: Ventricular zone
CP: Cortical plate
MZ: Marginal zone
EGFR: Epidermal growth factor receptor
CPC: Cortical progenitor cells
AD: Alzheimer’s disease
POA: Preoptic area of hypothalamus
